# A short-term educational program improved physicians’ adherence to guidelines for COPD and asthma in Shanghai

**DOI:** 10.1186/2001-1326-1-13

**Published:** 2012-07-03

**Authors:** Xiaocong Fang, Shanqun Li, Lei Gao, Naiqing Zhao, Xiangdong Wang, Chunxue Bai

**Affiliations:** 1 Department of Pulmonary Medicine, Research Institute of Respiratory Disease, Fudan University, Zhongshan Hospital, Shanghai, 200032, PR China; 2 College of public heath, Fudan University, Shanghai, 200032, PR China; 3 Biomedical Research Center, Zhongshan Hospital, Fudan University, Shanghai, 200032, PR China

**Keywords:** Chronic obstructive pulmonary disease, Asthma, Clinicians, Quality improvement

## Abstract

**Background:**

Chronic pulmonary diseases such as chronic obstructive pulmonary disease (COPD) and asthma exert increasing burden on society while the management of them is far from adequate. The objective of this study is to evaluate adherence to guidelines through a patient study, and then investigate the effects of a short-term quality improvement educational program among clinicians in Shanghai, China.

**Methods:**

A prescription survey was performed in a random sample of 100 COPD and asthma outpatients to assess their pharmacological therapy. Then, an educational program was conducted in young pulmonary physicians from 83 hospitals in Shanghai. The training course was divided into 7 sessions of 2 hours delivered over 4 days from July 2010 to August 2011. Three months later, all of the participants were asked to take a written examination to assess the efficiency of training.

**Results:**

Prescription survey among the patients indicated the prescriptions are not consistent with the recommendations of current GOLD and GINA guidelines. The mainly existing issue is the overuse of inhaled glucocorticosteroid. For the educational program, 161 pulmonary physicians have attended the training course, and 110 clinicians finished the tests with an attendance rate of 68.3%. Although most of the clinicians recognized the increasing burden of COPD and asthma, they do not know well about the core elements of guidelines and their clinical practice is not fully in agreement with current recommendations. Through crossover comparison, our results suggested clinicians’ knowledge of the guidelines was improved after training.

**Conclusions:**

We concluded that application of continuous educational programs among physicians might promote their adherence to guidelines, and by that improve the quality of healthcare.

## Background

Chronic obstructive pulmonary disease (COPD) and asthma are both chronic respiratory diseases characterized by the impairment of lung function, and they are also complex multi-component diseases accompanied with mental and physical co-morbidities [1,2]. In recent years, there has been increasing evidences suggesting that COPD and asthma are imposing enormous burden on patients, healthcare professionals and society in terms of morbidity, mortality, healthcare resources utilization and expense worldwide [3], especially in developing countries [4-9].

Despite these striking statistics, the management of COPD and asthma is far from adequate. One major barrier to better patient outcomes is under-diagnosis and under-treatment of the diseases. It was reported that only 1/3 of the patients were diagnosed early and that less than one-third of COPD diagnoses were supported by spirometry in China [9]. Give COPD and asthma are preventable and treatable diseases, clinical practice guidelines are systematically developed to improve the quality of care by providing information and sound clinical recommendations to clinicians [10]. Since the late 1980s, many international and national COPD and asthma management guidelines in different languages have been published and disseminated [10,11], but they are not yet implemented appropriately [12-14]. Phanareth et al. performed a telephone survey in all 70 hospitals in Denmark before and after the publication of national guidelines to examine treatment behaviors of physicians. The authors found that treatment behavior was only moderately affected by national guidelines, with overall poor adherence to guideline recommendations [15]. The same phenomenon was reported in Greece [16], Texas [17], and some European countries [18,19]. However, the level of implementation of guidelines in China is unclear and no relevant study has been conducted.

In addition, continuing medical education (CME) has become a popular strategy for health care promotion worldwide. The underlying rationale for CME is that providers who are educated about the latest standards of care will make more informed diagnostic and treatment decisions, resulting in improved patient outcomes [20,21]. So far, few educational programs in diversified forms have shown some success in improving physicians’ knowledge and performance in clinical practice. While among these studies, limitations were exsited, e.g., participation was sometimes voluntary, the enrolled general physicians might not be representative and leading to selective bias [22-26], control group was not perfectly matched, evaluation was always subjective by comparing the numbers of satisfactory quality cases in the general physicians’ clinics, educational courses were not well organized and official, and educational program specially focus on the some aspects like spirometry. Furthermore, such educational programs had not yet been reported in China.

The present study was designed to evaluate the knowledge of primary care clinicians in Shanghai about current guidelines for the management of COPD and asthma through a study among the patients, as well as investigate the impact of a short-term educational program on knowledge and performance of clinicians.

## Methods

### Investigation among patients

The population of Shanghai is 23 million. The prevalence of COPD is about 12% in people older than 60 years of age, which account for 13.26% of the total population [27,28]. It means there are about 640 COPD patients in Shanghai. The prevalence of adult asthma is about 1.8%, which account for 83% of the total population [28,29]. Our investigation was carried out in ten hospitals (3 Tertiary hospitals and 7 Secondary hospitals) from July 2010 to November 2010. Outpatients were selected randomly from asthma and COPD clinics. The inclusion criteria are: 1) over 40 years of age; 2) diagnosed with COPD or asthma according to recommendations of the Global Initiative for Chronic Obstructive Lung Disease (GOLD) and the Global Initiative for Asthma (GINA) [1,2]; 3) with a regular follow-up in respiratory clinics longer than 6 months. Excluded criteria are: 1) patients with diseases affecting the lung function or unable to complete spirometry test; 2) patients with co-morbidities which affect the pharmacological therapy of COPD or asthma; 3) unable to provide an intact medical history for at least 6 months. Informed consent was obtained for each patient. The protocol was approved by the ethics committee in Zhongshan Hospital, Fudan University. A total of 100 patients (43 COPD patients, 57 asthma patients) in these 10 hospitals were agreed as well as eligible to participate in our investigation. Although the participants were a convenience sample and not necessarily representative of the overall health care status for COPD and asthma patients in Shanghai, they were likely to be similar to other patients who engaged in a regular clinical visit in secondary or tertiary hospitals thus their health care records may reflect the management of COPD and asthma in these hospitals.

Information was obtained from both their medical records and the face-to-face interview regarding the socio-demographic characteristics, comorbidity, smoking habits, body mass index, years since diagnosis and current symptoms. The severity of airflow obstruction was assessed by spirometry. Their treatment prescriptions were evaluated preciously according to the GOLD and GINA guidelines by two senior general internal medicine clinicians and by one senior lung disease specialist, and then served as evidence to assess the pulmonary physicians’ adherence to guidelines.

### A short-term training course among clinicians

There are 19 administrative districts in Shanghai, with a total of 306 registered hospitals [30]. This session was carried out in 83 comprehensive hospitals in Shanghai, China, including 20 tertiary hospitals, 38 secondary hospitals and 25 primary hospitals from 19 administrative districts. All specialized hospitals, such as tumor hospitals, obstetrics and gynecology hospitals, pediatric hospital were excluded. A total of 641 pulmonary physicians in these 83 hospitals were listed as candidates. Inclusion criteria include: 1) pulmonary physicians; 2) aged 20–35 years; 3) attendings or residents with 1–5 years of clinical experience were first considered. Excluded those: 1) aged > 35 years; 2) chief physicians or associate chief physicians; 3) physicians with clinical experience > 10 years; 4) physicians had been involved in other training courses in the past year or at the same time frame to our training course. At last, two or three qualified volunteers in each hospital were finally accepted in this study with a total population of 161.

**Figure 1:**
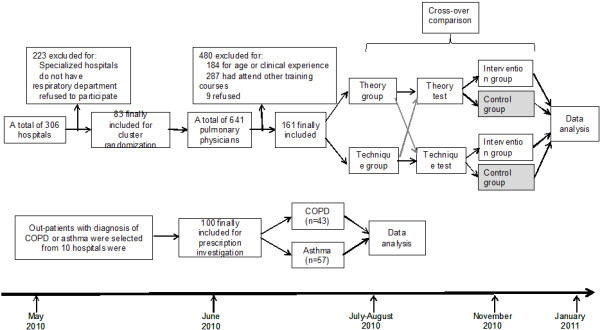
Flow diagram showing randomized controlled trial testing a quality improvement educational program in hospitals in Shanghai, China.

The intervention was divided into two parts: training and examination. The overall design of the study was shown in Figure 1. In the first part, all of the hospitals were divided into two groups by cluster-randomization according to their location (downtown or urban), grade (tertiary, secondary or primary) and properties (military or public) in order to avoid the bias due to their different baseline knowledge: (1) theory group and (2) technique group. The intervention consisted of a training seminar divided into 7 sessions of 2 hours delivered over 4 days from July 2010 to August 2010. During the intervention, clinicians in the theory group received the theory courses on COPD and asthma, while the technique group received the technique training. The component of training course provided to the two different groups were summarized in Table 1. Teaching documents, lecture notes, and reference texts were given to the participants. Physicians were also encouraged to implement what they have been told in the training courses into their clinical practices. The second part was conducted three months later, in which all of the participants were asked to take a written examination including both the theory and technique parts. Then when the data was statistically analyzed, the answers from physicians in the technique group or from physicians had not attend the training course (there are 12 physicians who had not attended the training course but participated the examination) were treated as control group in the theory part, and vice versa for the technique part.

**Table 1:** Curriculum for the quality improvement education program

Theory group	Theory courses
	· GOLD, Chinese guidelines for the prevention and management of COPD;
	· GINA, Chinese guidelines for the prevention and management of asthma;
	· Guideline for the diagnosis and treatment of Hospital Acquired Pneumonia;
Technique group	Technique courses
	· Clinical application and tips of lung function test;
	· Clinical application and tips of aerosol inhalation in the treatment of COPD and asthma;
	· Clinical application and importance of arterial blood gas test;
	· Clinical application and tips of mechanical ventilation.

The examination focused on five core elements of the guidelines: (1) epidemiology and identification of risk factors for COPD and asthma, (2) diagnostic criteria and procedures, (3) patient education and preventive measures, (4) pharmacological treatment strategies, (5) management of exacerbations. The education program and curriculum was developed by two pulmonary physicians (XCF and SQL), a staff from Shanghai quality control center of chronic lung diseases (LG) and a statistic specialist (NQZ), and then it was checked and approved by two senior pulmonary specialists (CXB and XDW) before being disseminated.

### Data analysis

The baseline characteristics of the patients were described using frequency tables for the nominal variables. The effectiveness of the educational intervention was assessed by comparing the answers of the questionnaire from the intervention group and control group. For the choice or multiple-choice questions, the answers were directly counted and analyzed by the different proportions from the two groups. For the essay questions (eg., the indications of long time oxygen therapy), the answers were quantified for every score point they answered, and then the graded into well, moderate and poor. Clinicians’ characteristics and answers were compared using the Fisher exact test or the Pearson Chi-Square test for proportions, weight cases by actual values of each group for multivariable. P ≤ 0.05 was considered statistically significant. Data were analyzed by SPSS 16.0 software.

## Results

### Assessment among the patients

One hundred patients (43 COPD patients, 57 asthma patients) participated in the prescription investigation. Table 2 shows baseline characteristics of all participants. Most patients had moderate or severe COPD, and mild persistent or moderate persistent asthma.

**Table 2:** Baseline characteristics of COPD and asthma patients

**Severity**	**Disease courses**	**Sex**^‡^		**Age (yr)**	**BMI**	**Smoking history (pack years)**	**FEV1/FVC**	**FEV1 predicted**
		**M**	**F**					
COPD*								
GOLD II	12.9 ± 11.2	10	2	69.7 ± 9.0	22.6 ± 4.6	27.5 ± 30.7	0.64 ± 0.1	0.63 ± 0.1
GOLD III	17.5 ± 14.7	21	0	73.6 ± 9.2	22.2 ± 3.4	33.0 ± 18.7	0.50 ± 0.1	0.39 ± 0.0
GOLD IV	13.6 ± 9.0	8	2	72.0 ± 6.5	19.9 ± 3.1	25.0 ± 17.1	0.54 ± 0.1	0.34 ± 0.2
**Asthma**†								
GINA I	1.5 ± 1.3	1	4	50 ± 19.4	22.4 ± 2.7	0 ± 0	0.70 ± 0.2	0.74 ± 0.2
GINA II	12.9 ± 15.8	8	12	51.2 ± 11.3	23.4 ± 5.0	5.2 ± 1.3	0.67 ± 0.3	0.83 ± 0.3
GINA III	17.8 ± 13.7	9	13	46.3 ± 17.8	22.7 ± 2.8	22.2 ± 6.7	0.65 ± 0.2	0.68 ± 0.3
GINA IV	11.7 ± 7.1	5	5	55.7 ± 18.2	22.7 ± 2.8	21.2 ± 10.1	0.63 ± 0.2	0.69 ± 0.2

* GOLD I: mild, GOLD II: moderate, GOLD III: severe, GOLD IV: very severe;

† GINA I: Intermittent, GINA II: Mild persistent, GINA III: Moderate persistent,GINA IV: Severe persistent.

^‡^ M: male; F: female.

In our investigation, most of the moderate COPD patients (75.0%, 9/12) were prescribed short acting bronchodilators, and 50.0% (6/12) of them were prescribed 2 or more different bronchodilators. The other most frequently prescribed medicines were combined long-acting β_2_ agonist (LABA) with steroid (58.3%, 7/12), antibiotics (58.3%, 7/12) and expectorants (50.0%, 6/12) (Table 3). Furthermore, theophylline was the most often prescribed bronchodilator (prescribed for 74.4% of COPD patients and 49.1% of asthma patients, data not shown) while it is not recommended as the first choice because of its potential toxicity [1]. The prescription for severe and very severe COPD patients was similar to that for moderate, except for an increased use for antitussive drugs among severe COPD patients (47.6%). The use of Chinese traditional medicine was increased as the disease progressing: 8.3% (1/12) in GOLD stage II patients, 19.1% (4/21) in GOLD stage III patients and 20.0% (2/10) in GOLD stage IV patents. However, Chi-Square test showed there is no significant linear-by-linear association (P = 0.448), which may partly due to the small sample size. In addition, 14.3% of the severe COPD patients were prescribed an oral corticosteroid on a chronic basis.

**Table 3:** Prescribed medicines for COPD patients

	**GOLD II**	**GOLD III**	**GOLD IV**	**Total**
	**(n = 12)**	**(n = 21)**	**(n = 10)**	**(n = 43)**
Short acting bronchodilator	75.0%	80.9%	80.0%	79.1%
Combined LABA with steroid	58.3%	61.9%	70.0%	62.8%
Antibiotics	58.3%	61.9%	60.0%	60.5%
Expectorants	50%	52.4%	70.0%	55.8%
Combined ≥2 bronchodilators	50.%	76.2%	70.0%	67.4%
Long acting bronchodilator	16.7%	9.5%	20.0%	14.0%
Anti-tussive	8.3%	47.6%	10.0%	27.9%
Chinese traditional medicine	8.3%	19.1%	20.0%	16.3%
Oral corticosteroids	0.0%	14.3%	0.0%	7.0%
ICS	0.0%	0%	10.0%	2.3%

The most often prescribed medicine for intermittent asthma was combined inhaler of LABA with steroid (80.0%, 4/5), followed by inhaled glucocorticosteroid (ICS) (60.0%, 3/5) and short acting bronchodilator (60.0%,3/5), leukotriene receptor antagonist (60.0%,3/5) and antibiotics (40.0%,4/5). For the mild persistent asthma, the most often prescribed medicines are: combined inhaler of LABA with steroid (57.9%, 11/19), ICS (52.6%, 10/19), leukotriene receptor antagonist (42.1%, 8/19), short acting bronchodilator (36.8%, 7/19), antibiotics (36.8%, 7/19), combined ≥2 bronchodilators (31.6%, 6/19) and oral corticosteroid (31.6%, 6/19). For the moderate persistent asthma patients, the 3 top prescribed medicines are short acting bronchodilator (71.4%, 15/21), antibiotics (52.4%, 11/21) and ICS (42.9%, 9/21). For the severe persistent asthma, 90.0% (9/10) of the patients were prescribed short acting bronchodilators, of whom 60.0% used 2 or more kinds of these drugs, followed by ICS (70.0%, 7/10) and antibiotics (70.0%, 7/10). The use of expectorants was increased (20.0% (1/5) in GINA I, 21.1% (4/19) in GINA II, 28.6% (6/21) in GINA III and 50.0% (5/10) in GINA IV, Chi-square tests linear-by-linear association = 0.713) while antitussive drugs decreased with the increasing severity of disease (20.0% (1/5) in GINA I, 10.5% (2/19) in GINA II, 9.52% (2/21) in GINA III and 0.0% (0/10) in GINA IV, Chi-square tests linear-by-linear association = 0.229) (Table 4).

**Table 4:** Prescribed medicines for asthma patients

	**GINA I**	**GINAII**	**GINA III**	**GINA IV**	**Total**
	**(n = 5)**	**(n = 20)**	**(n = 22)**	**(n = 10)**	**(n = 57)**
Combined LABA with steroid	80.0%	57.9%	19.1%	30.0%	38.6%
ICS	60.0%	52.6%	42.9%	70.0%	50.9%
Short acting bronchodilator	60.0%	36.8%	71.4%	90.0%	59.7%
leukotriene receptor antagonist	60.0%	42.1%	23.8%	40.0%	35.1%
Antibiotics	40.0%	36.8%	52.4%	70.0%	47.4%
Expectorants	20.0%	21.1%	28.6%	50.0%	28.1%
Chinese traditional medicine	20.0%	5.3%	14.3%	10.0%	10.5%
Antitussive	20.0%	10.5%	9.5%	0.0%	8.8%
Combined ≥2 bronchodilators	0.0%	31.6%	4.8%	60.0%	24.6%
Oral corticosteroid	0.0%	31.6%	28.6%	30.0%	26.3%

### Characteristics of clinicians

In the training course, a total of 641 pulmonary physicians were included in the candidate list, of whom 184 were excluded for age or clinical experience, 287 were excluded for having received other related training courses in the past year. Of the remained 170 eligible clinicians, 9 refused to attend the training with other personal reasons, leaving 161 in the final program with the allocation ratio of 25.1%. Among these enrolled clinicians, 75 were assigned to the theory group and 86 to the technique group by cluster randomization.

In the examination part, one hundred and ten clinicians attended the test and finished the questionnaire as demanding: including 49 from theory group (attendance rate 65.3%), 49 from technique group (attendance rate 57.0%) and 12 clinicians who did not participate in any of the training courses. For the theory part, 49 clinicians from theory group formed the intervention group, while the other 61 clinicians as a control group, and vice versa for the technique part. Thus, for both the theory and technique parts, there were 49 members in the intervention group and 61 in the control group. Clinicians’ characteristics for both groups are summarized in Table 5.

**Table 5:** Physician and practice characteristics (n = 110)

**Demographic variable**	**Theory part**			**Technique part**		
	**Intervention**	**Control**	**P**	**Intervention**	**Control**	**P**
	**N = 49**	**N = 61**		**N = 49**	**N = 61**	
Grade of hospital			NS			NS
Tertiary hospitals	11(22.4%)	19(31.1%)		13(26.5%)	17(27.9%)	
Secondary hospital	37(75.5%)	39(63.9)		34(69.4%)	42(85.7%)	
Community hospital	1(2.0%)	3(4.9%)		2(4.1%)	2(3.3%)	
Sex			NS			NS
Female	34(69.4%)	39(60.9%)		35(71.4%)	38(62.3%)	
Male	15(30.6%)	22(36.1%)		14(28.6%)	23(37.7%)	
Seniority in hospital			NS			NS
≤5y	28(57.1%)	27(44.3%)		21(42.9%)	34(55.7%)	
>5y	21(42.9%)	34(55.7%)		28(57.1%)	27(44.3%)	
Education			NS			NS
Bachelor	3(6.1%)	2(3.3%)		1(2.0%)	4(6.6%)	
Master	9(18.4%)	17(27.9%)		14(28.6%)	12(19.7%)	
Doctor	37(75.5%)	42(85.7%)		34(69.4%)	45(73.8%)	

### Theory of COPD

Most of the clinicians overestimate the prevalence of COPD and asthma in this survey (Table 3). Unanimously 91.8% (45/49) of the physicians in the intervention group and 95.1% (58/61) in the control group identify cigarette smoking as the most important risk factor for COPD (P = 0.76), the others considered infection is the most risk factor. While the answer for second risk factor was diversified, including occupational exposures (40.8% (20/49) vs. 34.4% (21/61)), infection (28.6% (14/49) vs. 45.9% (28/61)), solid fuel use (16.3% (8/49) vs. 16.4% (10/61)), smoking (6.1% (3/49) vs. 3.3% (2/61)), genetic susceptibility (2.0% vs. 0%) and social economic status (4.1% vs. 0.0%). There were no significant differences between the two groups (P = 0.25). Although GOLD guidelines recommend spirometry as the gold standard for diagnosis, only 89.8% (44/49) and 86.9% (53/61) of respondents from the intervention group and the control group respectively consider spirometry as the most appropriate test when a diagnosis of COPD is suspected. Despite the awareness of its importance, almost half of the clinicians (42.8% (11/49) in the theory group and 47.6% (21/61) in the technique group) make a diagnosis of COPD based on symptoms and medical history rather than spirometry (Table 6).

**Table 6:** Different answers to the knowledge of COPD between intervention group and control group

	**Intervention**	**Control**	**P value**
	**Group(n = 49)**	**Group(n = 61)**	
Prevalence of COPD in China			NS
Average of reported prevalence	11.2%	13.6%	
Average of reported prevalence	0.2–50.0%	0.2–70.0%	
Most important risk factors of COPD (% of respondents)			NS
Smoking	91.8%	95.1%	
Infection	8.2%	4.9%	
Most valuable indication for early diagnosis of COPD (% of respondents)			NS
Symptoms	16.3%	18.0%	
Physical examination	2.0%	8.2%	
Spirometry	89.8%	86.9%	
Chest x-ray	4.1%	8.2%	
First choice exam for confirming diagnosis (% of respondents)			NS
Spirometry	55.1%	54.1%	
Medical history (chronic bronchitis or emphysema)	22.4%	23.0%	
Symptoms	20.4%	24.6%	
First-line prescribed medicine for COPD			0.009
Beta_2_-agonists	49.0%	23.0%	
Combined beta_2_-agonists with corticosteroid	30.6%	42.7%	
Inhaled corticosteroid	8.2%%	11.8%	
Anticholinergic	4.1%	11.0%	
Theophylline	6.1%	8.2%	
First-line prescribed medicine for COPD exacerbation (% of respondents)			0.004
Antibiotics	42.9%	31.1%	
Oral corticosteroid	14.3%	21.3%	
Combined beta_2_-agonists with corticosteroid	14.3%	19.7%	
Beta_2_-agonists bronchodilators	24.5%	11.5%	
Anticholinergic agents	2.0%	3.3%	
Theophylline	0.0%	4.9%	
Provide smoking cessation counseling			NS
Always	97.0%	98.0%	
Occasionally	3.0%	2.0%	
Suggestions for taking influenza vaccine			0.03
Always	63.3%	47.5%	
Occasionally	36.7%	52.5%	
Indications for inhaled corticosteroid			NS
Know well	40.8%	27.9%	
Know moderately	26.6%	39.3%	
Know poor	32.6%	32.8%	

Short acting β_2_-agonists (SABA) or LABA were the first-line prescribed medications in 49.0% (24/49) and 23.0% (14/61) of physicians for COPD in the two groups (P = 0.009). Thirty percent (15/49) and 37.7% (23/61) of respondents prescribed a combined LABA and ICS, while 8.2% (4/49) and 9.8% (6/61) of them preferred ICS which is not recommended for stable COPD patients [31]. Nearly 60–70% of the clinicians ignore the accepted indications for the use of combination inhalers (Table 6).

For the management of acute exacerbation, the top three prescribed medicines were antibiotics (42.9% (21/49) in the theory group vs. 31.1% (19/61) in technique group), systemic corticosteroids (14.3% (7/49) vs. 21.3% (13/61)), combined inhaler of LABA and steroid (14.3% (7/49) vs. 19.7% (12/61)). There is no significant difference between the two groups (P = 0.527). SABA are usually the preferred bronchodilator. Almost all of the physicians (77.6% (38/49) in theory group vs. 82.0% (50/61) in technique group) report that they always provide smoking cessation counseling in clinic (P = 0.565). Only 63.3% (31/49) clinicians from the intervention group and 47.5% (39/61) from the control group recommend patients take influenza vaccines (P = 0.942) (Table 6).

### Theory of asthma

Seventy six percent (37/49) of the physicians in the intervention group and 57.2% (35/61) in the control group considered chronic inflammation associated with airway hyper-responsiveness being the physiological and pathological basis for asthma. Seventy-five (37/49) of the physicians in intervention group and 52.6% (32/61) of the clinicians reported that they are familiar with Chinese guidelines, while only 38.6% (19/49) and 22.9% (14/61) of the clinicians in each group were familiar with GINA. However only 44.9% (22/49) and 29.5% (18/61) of clinicians were able to classify disease severity in accordance with GINA criteria, 14.3% (7/49) and 24.6% of them mastered it moderately, while still 26.5% (13/49) and 45.9% (28/61) of them mastered it poor. (P = 0.049) (Table 7).

**Table 7:** Different answers to the knowledge of asthma between intervention group and control group

	**Intervention**	**Control**	**P value**
	**group(n = 49)**	**group(n = 61)**	
Basic pathology of asthma			0.000
Bronchial hyper-responsiveness	14.1%	29.5%	
Chronic inflammation	75.7%	57.2%	
Allergic disease	10.2%	13.3%	
The prevalence of asthma in China			NS
Average of reported prevalence	7.3%	7.3%	
Range of reported prevalence	0.5–35.0%	0.5–20.0%	
Declared familiarity with asthma guidelines			0.000
Not familiar with any	5.8%	16.3%	
International guidelines, GINA	38.6%	22.5%	
Chinese guidelines	75.6%	51.2%	
Other guidelines	22.1%	9.8%	
Knowledge for grading severity			0.049
Well	44.9%	29.5%	
Moderately	14.3%	24.6%	
Poor	26.5%	23.0%	
First-line prescribed medicine for mild asthma			0.005
Combined beta_2_-agonists with corticosteroid	44.3%	34.7%	
Beta_2_-agonists bronchodilators	14.8%	34.7%	
Inhaled corticosteroids	27.9%	26.5%	
Oral corticosteroids	6.6%	0.0%	
Leukotriene modifiers	4.9%	0.0%	
Are you using PEFM for follow up of your asthma patient?			NS
Yes	63.3%	60.7%	
No	8.2%	19.7%	
Depends on the patients’ condition	28.6%	19.7%	
Are you satisfied with the symptom control of your patients?			NS
Satisfied	10.2%	4.9%	
Basically satisfied	85.7%	85.2%	
Dissatisfied	4.1%	9.8%	
Do you use specific immunotherapy for allergic asthma?			0.009
Never	40.9%	59.0%	
Sometimes	46.9%	41.0%	
Always	12.2%	0.0%	
What’s the percent of patients who will fully follow your prescription?			NS
91–100%	4.1%	4.9%	
50–90%	75.5%	65.6%	
10–49%	20.4%	26.2%	
<10%	0.0%	3.3%	
What do you think are the major reasons for the lack of adherence among patients?			NS
Side effects related to therapy	16.3%	23.0%	
Unawareness of the benefits for pharmacotherapy	44.9%	47.5%	
Inconvenience	6.1%	11.5%	
Cost for pharmacotherapy	18.4%	6.6%	
Incorrect use of inhalation device	8.2%	11.5%	
Do you think such training course for clinicians is useful?			NS
Very useful	100.0%	98.4%	
Maybe useful	0.0%	1.6%	
Not necessary	0.0%	0.0%	
Do you think patient education is necessary?			NS
Quite necessary	100.0%	100.0%	
Necessary	0.0%	0.0%	
Not necessary	0.0%	0.0%	

Inhaled corticosteroids were reported as the first-line pharmacological therapy by 28.5% (14/49) and 26.3% (16/61) of the clinicians (P = 0.784), while 14.3% (7/49) and 34.5% (21/61) of them preferred LABA alone (P = 0.011). Twelve percent of the clinicians (6/49) in the intervention group would use specific immunotherapy for allergic asthma, which was significantly higher than that in the control group (none in 61 physicians) (P = 0.009). Most of the clinicians reported that more than 50.0% of the patients would adhere to their prescriptions and 85.0% of the clinicians were basically satisfied with the symptom control of their patients. Peak expiratory flow measurement (PEFM) was used by 63.3% (31/49) of the physicians in the theory group and 60.7% (37/61) in the technique group (P = 0.779). Clinicians reported that the major reasons for the lack of adherence included side effects of pharmacologic therapy, cost for drugs, and patients’ unawareness of the potential benefit of pharmacotherapy (Table 7).

### Technology

Spirometry is a useful tool to help confirm the diagnosis when it is suspected in a specific patient. However, there are still 4.1% (2/49) and 18.1% (11/61) of the clinicians did not use it to assess disease control (P = 0.024). Only 63.3% (31/49) and 60.5% (37/61) of the physicians in each group could define spirometric classification of COPD well, and 20.4% (10/49) of the physicians in the intervention group and 36.0% (22/61) in the control group could hardly master it at all (P = 0.025) (Table 8). When refer to the spirometric criteria for asthma diagnosis, only 34.7% (17/49) in the intervention group and 24.6% (15/61) in the control group could grasp the key points of the guidelines well (P = 0.096). The application of long-term oxygen therapy (>15 h/d) is extremely underused in Chinese patients with COPD. Only 67.4% (33/49) and 47.5% (29/61) of the clinicians reported they are familiar with the indications of long-term oxygen therapy (P = 0.111). Noninvasive mechanical ventilation (NIV) is also inappropriately used as only 40.8% (20/49) of the physicians in the intervention group and 37.7% (23/61) in the control group mastered the indications well, as high as 24.5% (12/49) of the physicians in the intervention group and 42.6% (26/61) in the control group mastered it really poor (P = 0.083).

**Table 8:** Different answers to the knowledge of technology and test between intervention group and control group

	**Intervention**	**Control**	**P value**
	**group(n = 49)**	**group(n = 61)**	
Do you use spirometry to confirm a suspected COPD?			NS
Yes	95.9%	100.0%	
No	4.1%	0.0%	
Do you use spirometry to assess the symptom control of your patients?			0.024
Yes	95.9%	81.9%	
No	4.1%	18.1%	
What do you think are the useful tests for confirming atypical asthma?			NS
Routine spirometry	38.8%	24.6%	
Airway responsiveness test	69.4%	55.7%	
Bronchodilator test	83.7%	83.6%	
PEF variation in 24 hours	71.4%	65.6%	
When do you prescribe aerosol inhalation for patients?			NS
Acute exacerbation	28.6%	19.7%	
Usual time	89.8%	82.0%	
When patients feel uncomfortable with other inhalation devices	16.3%	24.6%	
Knowledge of GOLD spirometric criteria for grading COPD			0.025
Know well	63.3%	60.5%	
Know moderately	16.3%	3.3%	
Know poor	20.4%	36.0%	
Knowledge of GINA spirometric criteria for the diagnosis of asthma			NS
Know well	34.7%	24.6%	
Know moderately	57.1%	52.3%	
Know poor	8.2%	23.1%	
Indications for noninvasive mechanical ventilation			NS
Know well	67.4%	47.5%	
Know moderately	26.5%	44.3%	
Know poor	6.1%	8.2%	
Indications for noninvasive mechanical ventilation			NS
Know well	40.8%	37.7%	
Know moderately	34.7%	19.7%	
Know poor	24.5%	42.6%	

## Discussion

**Figure 2:**
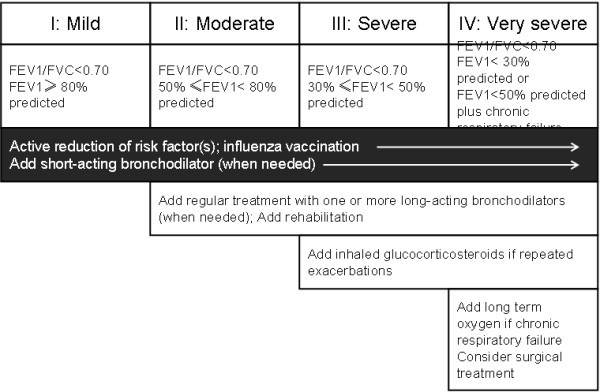
**Management of COPD according to the disease stage.** Cited from GOLD 2007 updated [32].

The results of the observational study among patients indicate that the prescribing practices of clinicians in Shanghai were not fully compliant with the recommendations of the GOLD and GINA guidelines. Depending on the severity of the disease and symptoms, GOLD guidelines recommend pharmacologic treatment as follows: as needed short-acting β_2_-agonists and anti-cholinergics for mild disease, and then a step-wise approach based on disease severity, including long-acting bronchodilators, with or without theophylline and (or) ICSs (Figure 2) [32]. It was stressed that, inhaled corticosteroids were, at the time of this study, only recommended to patients with severe and very severe COPD experiencing repeated exacerbations. From the prescription survey among the patients in our study, Table 3 showed that combined inhaler of LABA and corticosteroids were prescribed for 58.3% of the patients with moderate COPD, which is comparable to figures reported by other studies that inhaled corticosteroid is used in 60%–76% of the patients with mild stable COPD [22,33,34]. While one of the prominent feature found in our survey is the widely usage of combined inhaler of LABA and corticosteroid in patients at all stages which may partly due to the availability of the drugs. In contrast, short-acting bronchodilator, which is recommended as the cornerstone of pharmacological therapy, is only prescribed for 75% of the patients.

**Figure 3:**
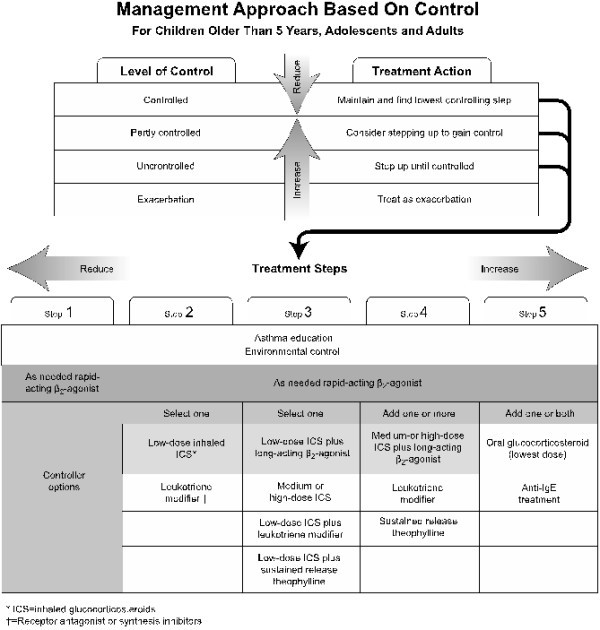
**Management approach for asthma based on control for children older than 5 years, adolescents and adults.** Cited from GINA 2010 updated [33].

For asthma, anti-inflammatory therapy is the cornerstone of pharmacotherapy for persistent asthma. International guidelines recommend the appropriate dose of ICS as the preferential choice. For children older than 5 years with worsening symptoms, options for controller therapy include increasing the ICS dose or add-on therapy with inhaled LABA or leukotriene receptor antagonist [2,33] (Figure 3). In our survey, only 60.0% of intermittent, 52.3% of the mild persistent, 42.9% of the moderate persistent and 70% of the severe persistent asthma patients were prescribed with ICS. However, it’s still much higher than that in Asian-Pacific study in 2003, which found that only 13.6% of respondents reported a current use of ICS [34]. While 31.6% of the mild persistent, 28.6% of the moderate persistent asthma patients were prescribed with oral corticosteroid that was only recommended for severe persistent asthma. ICS plus LABA was recommended for step 3 or upper stage therapy, while it had been prescribed for 80% of the intermittent asthma patients. Leukotriene modifiers was recommended as the alternative controller medications for patients who are unable or unwilling to use ICS, or who experience intolerable side effects was prescribed for 60.0% of the intermittent, 42.1% of the mild persistent, 23.8% of the moderate persistent and 40.0% of the severe persistent asthma patients in our survey.

Furthermore, through the examination among the physicians, we concluded that clinicians do not know well about the core elements of these guidelines and that their clinical practice is not in agreement with current recommendations. Although physicians recognize the importance of the problem [8,35], most of them do not use the recommended preventive measures (influenza vaccines), do not know well about the diagnostic criteria for COPD and asthma, and poorly recognize the indications and the benefits of the treatments they give to their patients. All of these may be partly explained by the lack of availability of the drugs, training in techniques and the poor socioeconomic standard of the patients. However, the study was conducted in Shanghai where should represent the highest quality of health care in China. Our results were consistent with previous studies conducted in China that have shown a lack of adherence with guidelines for COPD and asthma [36-39]. In addition, most of the clinicians noted that the major reasons for the lack of adherence by patients were they did not understand the benefits of therapy. Under this consideration, health education on self-management for patients should be taken into account to reduce the exacerbations and improve quality of life [40,41].

The education program in the present study included information on the evidence-based recommended pharmacological and non-pharmacological treatment, and in accordance with this we observed an improvement in most relevant indices. For example, after the training course, more physicians prefer to prescribed beta-2 agonists as the first-line prescribed medicine for COPD, while the use of ICS and combined inhaler of LABA and ICS was reduced. Besides, more physicians in the intervention group would suggest patients taking influenza vaccine as non-pharmacological therapy to reduce acute exacerbation (Table 6). For asthma, more physicians in the intervention group reported more familiar with asthma guidelines, asthma control evaluation as well as the use of specific immunotherapy for allergic asthma (Table 7). The technological training course focused on the application of spirometry, long-term oxygen therapy of COPD and noninvasive mechanical ventilation. According to GOLD guidelines, spirometry is the gold standard in the measurement of airflow limitation^1^ because of its reliable, simple, safe and inexpensive grading, monitoring, and assessing of the disease. However, it was reported that less than one-third of COPD diagnoses were made with the aid of spirometry in China [42,43]. The reported application rate of spiromery for COPD diagnosis was as high as 100% in our study even in the control group. However, physicians in the intervention group reported better grasp the spirometric criteria for disease diagnosis (Table 8). After the training course, 95.1% of the physicians reported would use spirometry to assess the symptom control of COPD or asthma versus 81.6% of the control group (P < 0.05). Likewise, long-term oxygen therapy and noninvasive mechanical ventilation were always underused in for patients met the criteria [44]. In our study, physicians in the intervention group were also more familiarized with the indications for long term oxygen therapy and noninvasive mechanical ventilation.

It’s obvious that our study showed clinicians’ knowledge of the guidelines was improved after the training curriculum. However, it should also be noted that the observed effects were smaller than that reported by other studies [22-26]. There are various reasons, including the short term of the training course, the different educational environment in different studies or the communication between the two groups thus leading to dissemination of knowledge. The main weakness of this study is the short time period which added difficulty for us to study the possible effect of the training course on the quality of patient care. Besides, no pre-test were performed in our study thus comparison between the pre-test and post-test could not be drawn. However, we did cluster randomization to divide these hospitals according to their location and grade in order to avoid the bias due to their different baseline knowledge.

## Conclusions

In conclusion, our study showed that the management chronic lung diseases, including pharmacological and non-pharmacological treatment, is not adherence to the current guidelines in Shanghai, but improvements can be achieved in most relevant indices of quality of care through educating the physicians. Further studies are needed in order to ascertain how the achieved improvements can be maintained and expanded.

In the future, governmental, regulatory, and accreditation bodies will become increasingly involved with ensuring that valid guideline recommendations find their way into clinical practice. We anticipate that COPD guideline recommendations will soon be incorporated into quality measures for hospitals and ambulatory practice settings and that those measures will be reported directly to the public.

It was reported that physicians prefer immediately available information that describe medication, disease classification, patient evaluation, treatment and monitoring. These preferences reflect the need to facilitate rapid decision making in the clinical setting [45]. These findings indicate that the contents of COPD or asthma guidelines should be summarized and disseminated as convenient and easily accessible tools, such as algorithms, flowcharts, or flow diagrams as pocket-sized cards.

## Abbreviations

COPD, Chronic obstructive pulmonary disease; CME, Continuing medical education; GOLD, Global Initiative for Chronic Obstructive Lung Disease; GINA, Global Initiative for Asthma; LABA, Long-acting β2 agonist; ICS, Inhaled glucocorticosteroid; SABA, Short acting β2-agonists; PEFM, peak expiratory flow measurement; NIV, Noninvasive mechanical ventilation.

## Competing interests

The authors have no conflicts of interest to disclose.

## Authors’ contributions

XF contributed to the organization of the training, acquisition of data, the study concept and design, analysis and interpretation of the data and drafting the manuscript. SL contributed to the acquisition of data, organization of the training, the study concept and design. LG contributed to the organization of the training, acquisition of data, the study concept and design. NZ contributed to the study design. XW contributed to critical revision of the manuscript. CB contributed to the study concept and design, acquisition of data, and critical revision of the manuscript. All authors read and approved the final manuscript.

## Funding/Support

The present work was supported by Shanghai Leading Academic Discipline Project (Grant B115); Chinese Medical Association Project (07010330041).
